# Survey of perspectives of people with inherited retinal diseases on ocular gene therapy in Australia

**DOI:** 10.1038/s41434-022-00364-z

**Published:** 2022-10-02

**Authors:** Heather G. Mack, Alexis Ceecee Britten-Jones, Myra B. McGuinness, Fred K. Chen, John R. Grigg, Robyn V. Jamieson, Thomas L. Edwards, John De Roach, Fleur O’Hare, Keith R. Martin, Lauren N. Ayton

**Affiliations:** 1 Ophthalmology, Department of Surgery, University of Melbourne, Melbourne, VIC Australia; 2 Centre for Eye Research Australia, Royal Victorian Eye and Ear Hospital, Melbourne, VIC Australia; 3 Department of Optometry and Vision Sciences, University of Melbourne, Melbourne, VIC Australia; 4 Centre for Epidemiology and Biostatistics, Melbourne School of Population and Global Health, University of Melbourne, Melbourne, VIC Australia; 5 Centre for Ophthalmology and Visual Sciences (incorporating Lions Eye Institute), The University of Western Australia, Perth, WA Australia; 6 Royal Perth Hospital and Perth Children’s Hospital, Perth, WA Australia; 7 Save Sight Institute, Faculty of Medicine and Health, The University of Sydney, Sydney, NSW Australia; 8 Eye Genetics Research Unit, Sydney Children’s Hospitals Network, Save Sight Institute, Children’s Medical Research Institute, University of Sydney, Sydney, NSW Australia; 9 The Australian Inherited Retinal Disease Registry and DNA Bank, Perth, WA Australia

**Keywords:** Diseases, Genetics

## Abstract

Many gene therapies are in development for treating people with inherited retinal diseases (IRD). We hypothesized that potential recipients of gene therapy would have knowledge gaps regarding treatment. We aimed to assess knowledge, attitudes, and perceptions of genetic therapies among potential recipients with IRD, using a novel instrument we designed (Attitudes to Gene Therapy-Eye (AGT-Eye)) and their associations with demographic data, self-reported visual status, and tools assessing quality of life and attitudes toward clinical trials using a community-based cross-sectional survey of Australian adults with IRD. AGT-Eye, overall quality of life EQ-5D-5L, National Eye Institute Visual Functioning Questionnaire (NEI-VFQ-25) and Patient Attitudes to Clinical Trials (PACT-22) instruments were administered. Six hundred and eighty-one people completed the study, 51.7% women of mean age 53.5 years (SD ± 15.8). Most participants (91.6%) indicated they would likely accept gene therapy if it was available to them or family members. However, only 28.3% agreed that they had good knowledge of gene therapy. Most obtained information about gene therapy from the internet (49.3%). Respondents with post-graduate degrees scored highest compared to other educational levels on methods (*p* < 0.001) and outcomes (*p* = 0.003) and were more likely to see economic value of treatment (*p* = 0.043). Knowledge gaps were present regarding methods and outcomes of gene therapy. This survey has shown high level of interest in the IRD community for gene therapies, and highlights areas for improved clinician and patient education.

## Introduction

Voretigene neparvovec-rzyl (“AAV2-hRPE65v2”, Luxturna®) has recently been approved by multiple global regulators for treatment of bi-allelic *RPE65* retinopathy in adults and children. It is the third gene-based therapy approved for human use [1, 2], and the first ocular gene replacement therapy [3]. Improvements in navigation, light sensitivity and visual field parameters are reported to be maintained up to 4 years post intervention [4]. At least 15 other inherited retinal diseases (IRDs) and optic nerve pathologies currently have gene therapy clinical trials underway [5].

Previous studies have shown that potential candidates for gene therapy trials have a desire to obtain knowledge about the treatment (information need), but can overestimate clinical effect and underestimate risk [6, 7]. Similarly in ophthalmology, potential participants in Phase I/2a gene therapy trials for X-linked retinoschisis required further information [8], and were motivated by therapeutic hope, although the authors considered the individuals to be realistic in their assessments [9]. Potential *RPE65* gene therapy recipients in the USA overestimated clinical effect (e.g., 4/10 wanted the ability to read print, an unlikely outcome) [10]. Even in standard treatments of retinal conditions (non-gene therapy), participants may have an incomplete understanding of the process [11].

Previous studies of patient expectations have focused on participants in clinical trials of gene therapies [8, 9]. Now that voretigene neparvovec-rzyl has been approved and made commercially available, it is important to gain an understanding of the community’s attitudes and perspectives on a regulatory-approved treatment (as compared to a clinical trial).

The objectives of this study were to assess knowledge, attitudes and perceptions of approved and future genetic therapies among Australians with (or a carer of someone with) an IRD. We sought to assess differences in these characteristics according to measures of quality of life, attitudes toward clinical trials, and vision-related quality of life, using a combination of novel and previously validated survey tools.

## Patients and methods

### Participants and recruitment

**Fig. 1:**
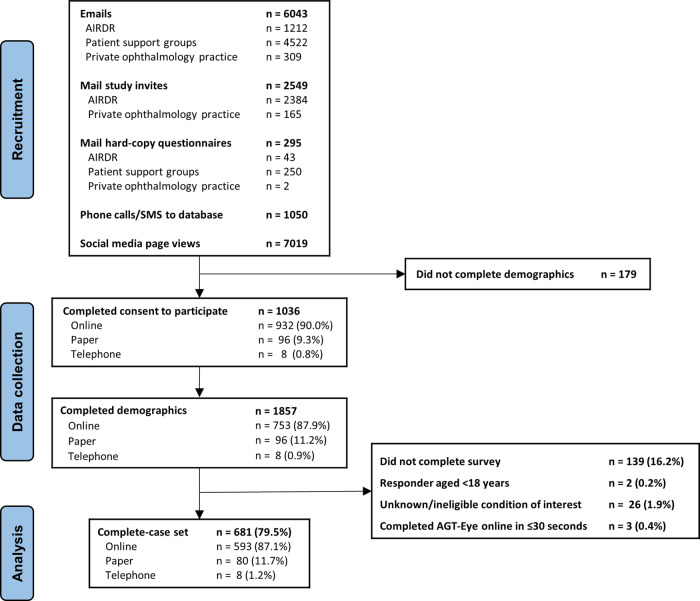
Participant flow chart. Participant flow chart showing recruitment, consent, method of survey answered and complete case set for an Australian national survey of people with IRD.

The detailed study protocol has been published elsewhere [12]. In brief, between 27 January 2021 and 7 June 2021, Australians with IRD, parents or caregivers of individuals under 18 years of age, and caregivers of adults with IRD were invited to participate in an online survey. The survey was hosted on REDCap (hosted at Centre for Eye Research Australia), a secure web application for building and managing online surveys and databases [13]. Recruitment avenues (Fig. 1) included mail, email, SMS, social media and/or telephone calls from patient support groups and private practices of authors with IRD clinics (HM, FC, JG). Fellows of the Royal Australian and New Zealand College of Ophthalmologists were notified and provided with a link to the survey to forward to their patients.

Participants self-selected their ocular diagnosis from a list of IRDs, and no independent confirmation of genotype or diagnosis was obtained. People who were carriers of IRD mutations without an ocular phenotype, or those with other polygenic retinal conditions with complex genetic risk factors (such as age-related macular degeneration) were excluded.

### Survey instruments and scoring

Demographic information collected included participants’ age and gender (and that of their child/dependent, if applicable), highest level of education, annual household income, number of members living in the household, primary IRD diagnosis, and details of first symptoms. Participants were also asked about their participation in medical research of any kind (including whether they had previously supplied DNA to any Australian IRD database), their likelihood of taking up gene therapy treatment if it was available to them, and their perceived barriers to receiving gene therapy.

Participants responded in sequence to 22 items in the Attitudes to Gene Therapy-Eye (AGT-Eye) survey [14]. As decision makers, parents/caregivers were asked to give their own response rather than provide the answer they believed their dependent would give. Responses were rated on a five-point Likert scale from 1 (Strongly disagree) to 5 (Strongly agree). Psychometric properties of AGT-Eye were previously investigated using item response theory methodology, resulting in four subscales used in this study: Sources of information, Knowledge of methods, Awareness of outcomes, and Perceived value of treatment [14].

The following validated survey tools were also included in the protocol: EQ-5D-5L Questionnaire [15], to assess overall quality of life using a utility score and visual analogue score (Australian self-complete version); National Eye Institute Visual Functioning Questionnaire (NEI-VFQ-25) [16], a patient-reported outcome instrument widely used in clinical trials; and the PACT 22 Clinical Trial Attitudes Scale [17] to assess attitudes toward clinical trial participation, including therapeutic misconceptions. Copyright owners of the three validated survey tools provided permission for their use. EQ-5D-5L and NEI-VFQ-25 were scored according to published methods [15–17]. For PACT-22, each question was scored between 1 and 5: 1 represents Strongly disagree and 5 represents Strongly agree. Subscale scores (Positive beliefs, Safety, Information needs, Negative expectations, Patient involvement) were calculated as the mean of item responses and standardized to a scale from 0 (high level of disagreement) to 100 (high level of agreement).

### Data capture and statistical analysis

Only participants with complete data on all instruments were included. Results of individuals with online completion of the AGT-Eye in ≤30 s were excluded from analysis as deemed unreliable (Fig. 1). Participants were classified as having either generalized or macular vision loss by their self-reported IRD diagnosis, according to an established protocol [14].

Participant characteristics were compared between respondent types (adults with IRD vs. parent/caregiver) using a two-sample *t*-test for normally distributed variables (age), Wilcoxon rank-sum test for continuous skewed variables (age of symptom onset and instrument scores) and Fisher’s exact test for categorical variables.

AGT-Eye items were collapsed to a three-point scale to provide a summary of individual AGT-Eye items (collapsing “Agree” and “Strongly agree” and “Disagree” and “Strongly disagree”), and evaluated using their original scores (scored from 1–5).

For evaluation of AGT-Eye subscale scores, AGT-Eye items 4, 6, 7, 8, 12, and 16 were reverse coded prior to scoring for consistency of score interpretation prior to averaging within subscales. Mean subscale scores range from 1–5. For subscales A (sources of information), higher scores indicate stronger agreement to having obtained information on gene therapy from different sources. For subscales B (knowledge of methods) and C (awareness of outcomes), higher scores indicate greater knowledge and awareness, respectively. For subscale D (perceived value), higher scores indicate a greater perceived value of having gene therapy treatment. Subscale scores were compared between participant characteristics via a two-sample t-test (respondent status, gender, and vision loss status) or ANOVA (likelihood of taking up gene therapy and educational level).

Correlations between instruments (AGT-Eye, NEI-VFQ-25, PACT-22, and EQ-5D-5L) were quantified using Spearman’s rank correlation coefficient. Statistical analyses were conducted using Stata/BE v17.1 (College Station, Tx).

## Results

### Participants

Approximately 9937 separate approaches were made to potential participants (Fig. 1), with a 5.3% click-through to the survey from recruiting emails. A social media campaign resulted in 7019 unique webpage views, 3.0% like or engagement rate, and a request for 37 mailed questionnaires. Participants may have been contacted several times by different organizations.

Consent was obtained for 1036 participants. After removal of ineligible responses, data from 681 participants (51.7% women, mean age 53.5 years (SD ± 15.8)) were available for analysis (Fig. 1). The majority of responses (87.1%) were completed online.

Based on a population frequency of 1 in 2000 individuals [18], Australia’s population of patients with IRD is currently estimated at 13,000 (both diagnosed and undiagnosed), resulting in a response rate of 5.3% of the target population.

**Fig. 2:**
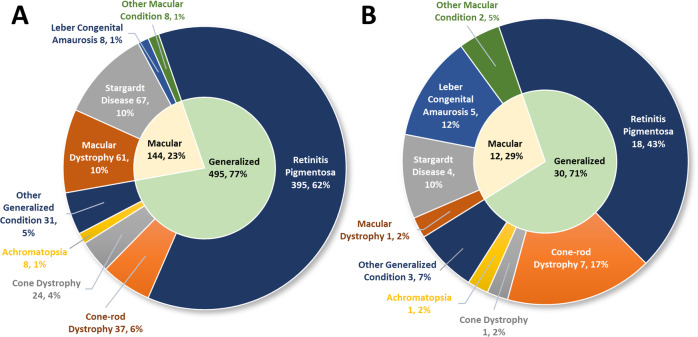
Self-reported diagnoses of respondents (*n* = 681). Self-reported diagnoses of respondents to national survey of Australians with inherited retinal disease **A** adults *n* = 639, **B** report of IRD for their dependent by caregivers *n* = 42.

Participant characteristics are shown in Table 1. Included adults with IRD (*n* = 639, 93.8%) were of mean age 54.1 years (SD 15.9, range 18–93), 50.2% female, and most had retinitis pigmentosa (61.8%; Fig. 2A). Responses from 42 (6.2%) parents/caregivers differed significantly from the adult cohort, being younger with mean age 44.5 years (SD 10.7, range 18–76), more likely to be female (73.8%) and disproportionately responding on behalf of individuals diagnosed with cone-rod dystrophy (16.7% compared to 5.8%) and Leber Congenital Amaurosis (11.9% compared to 1.3%, Fig. 2B).

**Table 1:** Participant characteristics in the Attitudes to Gene Therapy for the Eye Study.

	Respondent status		Total	*p* value^a^
	Adult patient	Parent/caregiver		
	(*n* = 639)	(*n* = 42)	(*N* = 681)	
Respondent age, years				**<0.001**
Range	18–93	18–76	18–93	
Mean (SD)	54.1 (15.9)	44.5 (10.7)	53.5 (15.8)	
Submission type, *n* (%)				0.677
Online	554 (86.7%)	39 (92.9%)	593 (87.1%)	
Paper	77 (12.1%)	3 (7.1%)	80 (11.7%)	
Phone interview	8 (1.3%)	0 (0.0%)	8 (1.2%)	
Gender, *n* (%)				**0.010**
Male	316 (49.5%)	11 (26.2%)	327 (48.0%)	
Female	321 (50.2%)	31 (73.8%)	352 (51.7%)	
Non-binary	2 (0.3%)	0 (0.0%)	2 (0.3%)	
Highest level of education completed, *n* (%)				0.728
Primary school	15 (2.3%)	0 (0.0%)	15 (2.2%)	
Secondary school (Year 10 or above)	209 (32.7%)	14 (33.3%)	223 (32.7%)	
Trade certificate	125 (19.6%)	5 (11.9%)	130 (19.1%)	
Bachelor’s degree	159 (24.9%)	13 (31.0%)	172 (25.3%)	
Post-graduate degree	117 (18.3%)	9 (21.4%)	126 (18.5%)	
I prefer not to say	14 (2.2%)	1 (2.4%)	15 (2.2%)	
Gross annual household income, *n* (%)				0.268
<$18,200	41 (6.4%)	1 (2.4%)	42 (6.2%)	
$18,201–$37,000	113 (17.7%)	3 (7.1%)	116 (17.0%)	
$37,001–$87,000	157 (24.6%)	11 (26.2%)	168 (24.7%)	
$87,001–$180,000	168 (26.3%)	17 (40.5%)	185 (27.2%)	
More than $180,001	57 (8.9%)	4 (9.5%)	61 (9.0%)	
I prefer not to say	103 (16.1%)	6 (14.3%)	109 (16.0%)	
Age when symptoms first appeared, years				**<0.001**
Range	0–81	0–40	0–81	
Median (IQR)	22 (11–36)	3 (0–6)	20 (10–35)	
Most recent decline in vision within, *n* (%)				**<0.001**
No decline, stable vision	60 (9.4%)	15 (35.7%)	75 (11.0%)	
Less than 6 months	79 (12.4%)	3 (7.1%)	82 (12.0%)	
1 year	130 (20.3%)	8 (19.0%)	138 (20.3%)	
5 years	216 (33.8%)	12 (28.6%)	228 (33.5%)	
10 years	154 (24.1%)	4 (9.5%)	158 (23.2%)	

*IRD* inherited retinal disease.

^a^*p* value from two-sample *t*-test (age), Wilcoxon’s rank-sum test (age of symptom onset), and Fisher’s exact test (categorical variables).

Bold values indicate *p* < 0.05.

Adults with IRD reported mean symptom onset age of 22 years (IQR 11–36, range 0–81); 9.1% reported they currently have stable vision, and 66.5% reported visual decline within the past 5 years (Table 1). As expected, parents/caregivers reported earlier symptom onset in their children/dependents (median 3 years, IQR 0–6, range 0–40; *p* < 0.001).

As expected, people with generalized retinal involvement were more likely to report difficulty seeing at night, bumping into low-lying objects, difficulty adjusting from light to dark (or vice versa), missing parts of vision or noticing peripheral or side vision reducing compared to those with macular disease (*p* < 0.05 for all comparisons; Supplementary Table S1). Patients with macular only involvement were more likely to report no noticeable symptoms.

Those with generalized retinal involvement were more likely to have previously taken vitamin A (generalized vs. macular: 17.5% vs. 7.1%; *p* = 0.001). Those with macular involvement were more likely to use herbal remedies (generalized vs. macular: 9.7% vs. 17.3%) for their IRD (Supplementary Table S1).

**Fig. 3:**
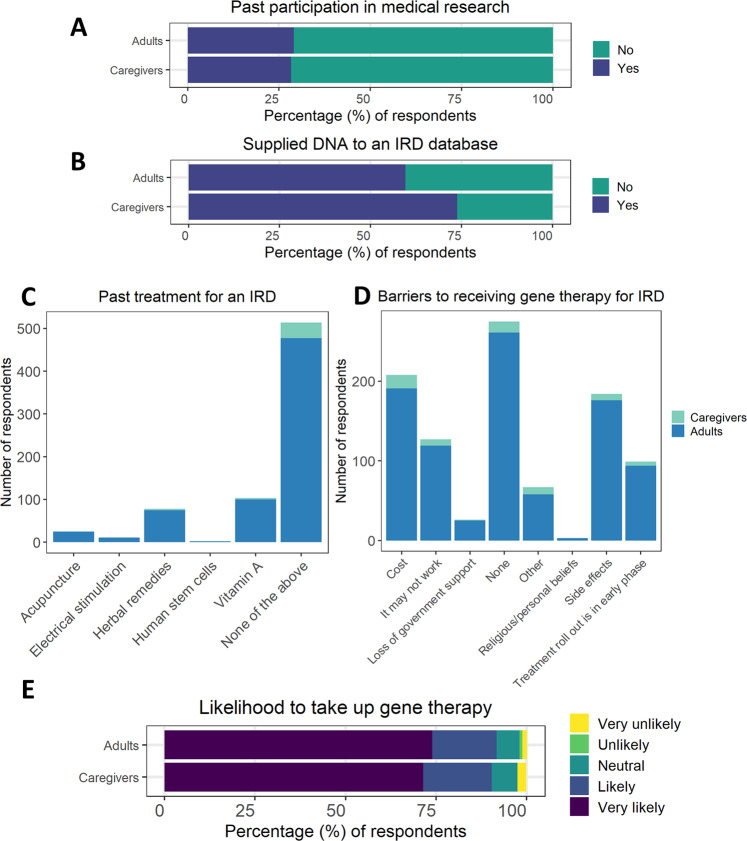
Attitudes to medical research of respondents (*n* = 681). Self-reported attitudes to medical research of respondents to national survey of Australians with inherited retinal disease (total *n* = 681, adults with IRD *n* = 639, parents/caregivers *n* = 42). **A** Previous participation in medical research of any kind, **B** previous supply of DNA to an IRD database, **C** previous treatment for IRD, **D** perceived barriers to receiving gene therapy for IRD, and **E** likelihood of consenting to gene therapy if offered for their condition. Responses for the two groups were not statistically different. IRD inherited retinal disease.

Overall, 28.3% of all respondents reported participation in medical research in the past (Fig. 3A), and 60.5% had supplied DNA to an Australian IRD database (Fig. 3B). Previous treatment for IRD was reported by 24.5% of respondents (Fig. 3C).

Overall, 59.6% of respondents reported one or more perceived barriers to receiving gene therapy (Fig. 3D). The commonest reported barriers were out of pocket cost (30.5%) and fear of side effects (27.0%). Despite these barriers, most participants (91.6%) indicated that they would likely (17.9%) or very likely (73.7%) take up gene therapy if it was available now to them or their family members for their IRD (Fig. 3E).

### AGT-Eye

Collapsed response frequencies to the AGT-Eye for all survey respondents are shown in Table 2, and full responses by respondent type (five-point Likert scale) are shown in Supplementary Fig. 1.

**Table 2:** Collapsed response frequencies to items of the AGT-Eye (*n* = 681).

Subscale	Item		Response, *n* (%)		
			Agree/strongly agree	Neither agree/disagree	Disagree/strongly disagree
	2	I have obtained information about gene therapy from			
A. Sources of information	2f	Internet	336 (49.3)	110 (16.2)	235 (34.5)
	2a	My ophthalmologist	258 (37.9)	122 (17.9)	301 (44.2)
	2c	Registry (e.g., Australian Inherited Retinal Disease Register)	187 (27.5)	136 (20.0)	**358 (52.6)**
	2d	Research group	162 (23.8)	145 (21.3)	**374 (54.9)**
	2i	Family/friends	149 (21.9)	154 (22.6)	**378 (55.5)**
	2b	Other medical or health professional	131 (19.2)	125 (18.4)	**425 (62.4)**
	2g	Social media	114 (16.7)	122 (17.9)	**445 (65.3)**
	2e	Newspapers	109 (16.0)	142 (20.9)	**430 (63.1)**
	2h	Patient support group	95 (14.0)	134 (19.7)	**452 (66.4)**
B. Knowledge of methods	1	I have good knowledge about gene therapy for inherited retinal diseases.	193 (28.3)	235 (34.5)	253 (37.2)
	3	I understand the difference between an experimental treatment provided in a clinical trial and a treatment that has already been approved by the Australian Government.	**592 (86.9)**	59 (8.7)	30 (4.4)
	4	*Gene therapy for the eye is suitable at any stage of a person’s life*.	60 (8.8)	272 (39.9)	349 (51.2)
	5	Generally, gene therapy for inherited retinal disease is delivered to both eyes	289 (42.4)	338 (49.6)	54 (7.9)
	6	*Gene therapy for the eye is injected into the blood stream through the arm*	171 (25.1)	**421 (61.8)**	89 (13.1)
	7	*Gene therapy and stem cell therapy are the same treatment*	321 (47.1)	308 (45.2)	52 (7.6)
C. Awareness of potential outcomes	8	*Gene therapy for the eye can restore vision back to normal*	257 (37.7)	**348 (51.1)**	76 (11.2)
	9	Gene therapy for the eye is a treatment that may slow down the disease	**472 (69.3)**	202 (29.7)	7 (1.0)
	10	Treatment complications to my eyes, such as permanent blindness, are possible with an approved gene therapy	216 (31.7)	**388 (57.0)**	77 (11.3)
	11	Gene therapy in my eye may have side effects elsewhere in my body	162 (23.8)	**416 (61.1)**	103 (15.1)
	12	*Having gene therapy for their eye condition means a person will not pass on an eye condition to any children they may have in the future*	**422 (62.0)**	220 (32.3)	39 (5.7)
	13	I may not be eligible for financial or other government benefits if my gene therapy for my eye condition is successful.	267 (39.2)	**345 (50.7)**	69 (10.1)
	14	Gene therapy for inherited retinal diseases will require many years of follow-up with my eyecare practitioner	**456 (67.0)**	207 (30.4)	18 (2.6)
	15	Receiving gene therapy for my inherited retinal disease means I won’t be eligible for future genetic treatments	37 (5.4)	**431 (63.3)**	213 (31.3)
	16	*I will lose my privacy if I undergo gene therapy, and my data will be in the public domain*	**479 (70.3)**	152 (22.3)	50 (7.3)
	17	If I undergo gene therapy, it will affect my eligibility or terms of conditions in life, disability or health insurance in the future	87 (12.8)	**368 (54.0)**	226 (33.2)
D. Value of treatment	18	The government should pay all costs of my gene therapy	292 (42.9)	275 (40.4)	114 (16.7)
	19	Government subsidy of my treatment would be an effective use of taxpayer money	**538 (79.0)**	126 (18.5)	17 (2.5)
	20	If gene therapy for my condition was not available in my state I would consider traveling interstate to access it	**521 (76.5)**	102 (15.0)	58 (8.5)
	21	My private health insurance should pay all out of pocket costs for my gene therapy	314 (46.1)	284 (41.7)	83 (12.2)
	22	I would consider a payment plan for my gene therapy.	**424 (62.3)**	196 (28.8)	61 (9.0)

AGT-Eye Items were asked in sequence from 1, 2a–2i, to 22. Questions for 2a–2i are listed from the highest level of agreement. Italicized items are reversed in score calculations for AGT-Eye subscale evaluations for consistency of interpretation. Items in bold indicates >50% of responses.

#### Individual items

The majority (86.9%) of participants agreed that they understood the difference between a clinical trial and an approved treatment. However, only 28.3% agreed that they had a good knowledge of gene therapy. Most participants reported obtaining information about gene therapy (Section A) from the internet (49.3%) and their ophthalmologist/s (37.9%).

Section B evaluated participants’ self-reported knowledge of gene therapy methods (Table 2). This subscale showed that most respondents were uncertain of the details of the treatment, with the most common response being “neither agree or disagree.” Almost half (47.1%) of respondents correctly indicated that gene therapy and cell therapy are not the same treatment, and 42.4% of respondents agreed that gene therapy for IRDs is generally delivered to both eyes.

Section C evaluated participants’ awareness of potential gene therapy outcomes (Table 2). Most respondents correctly indicated that gene therapy for the eye is a treatment that may slow disease progression (69.3%) and that gene therapy for IRDs will require many years of follow-up with their eyecare practitioner (67%). Most respondents also were aware that their privacy would be maintained if they received gene therapy (70.3%). Most were aware that having gene therapy for their eye condition could still mean passing their underlying genetic condition to future generations, depending on the mode of inheritance (62%).

Section D evaluated the perceived value of gene therapy treatment, including economic considerations. Although 79% of the respondents agreed that government subsidy of their gene therapy treatment would be an effective use of taxpayer money, only 42.9% of respondents indicated that the government, and 46.1% their private health insurance, should pay all costs of their gene therapy. Three quarters of respondents would consider traveling interstate to access gene therapy (76.5%), and 62% would consider a payment plan for their gene therapy.

#### Subscale quantitation and relationship with demographic parameters

Table 3 shows the mean scores across the AGT-Eye subscales, according to respondent characteristics. Subscale responses ranged from 2.4–3.7 (out of a maximum score 5), indicating uncertainty or neutrality of responses. There were no differences between the AGT-Eye subscale scores between males and females.

**Table 3:** AGT-Eye subscale scores according to respondent characteristics (*n* = 681).

	*n*	AGT-Eye subscale							
		Information sources		Methods		Outcomes		Value	
		Mean (SD)	*p*^a^	Mean (SD)	*p*^a^	Mean (SD)	*p*^a^	Mean (SD)	*p*^a^
Subscale score	681	2.43 (0.89)		3.29 (0.42)		3.37 (0.30)		3.74 (0.54)	
Respondent status									
Adult patient	639	2.42 (0.89)		3.28 (0.42)		3.37 (0.30)		3.72 (0.55)	
Parent/caregiver	42	2.64 (0.82)	0.115	3.39 (0.51)	0.099	3.36 (0.24)	0.862	3.99 (0.44)	**0.002**
Gender									
Male	327	2.44 (0.89)		3.31 (0.44)		3.37 (0.31)		3.70 (0.54)	
Female	352	2.42 (0.89)	0.771	3.27 (0.41)	0.291	3.37 (0.29)	0.874	3.77 (0.54)	0.106
Type of vision loss									
Widespread	525	2.45 (0.88)		3.27 (0.42)		3.38 (0.30)		3.73 (0.54)	
Macular	156	2.38 (0.93)	0.380	3.35 (0.44)	**0.048**	3.33 (0.30)	0.062	3.78 (0.55)	0.300
Likelihood of taking up gene therapy									
Unlikely/very unlikely	57	2.25 (0.89)		3.29 (0.53)		3.37 (0.33)		3.47 (0.59)	
Neutral	122	2.25 (0.82)		3.20 (0.36)		3.33 (0.28)		3.6 (0.47)	
Likely/very likely	502	2.50 (0.90)	**0.005**	3.31 (0.42)	**0.036**	3.38 (0.30)	0.296	3.81 (0.54)	**<0.001**
Highest level of education completed									
Primary school	15	2.07 (1.10)		3.03 (0.49)		3.19 (0.29)		3.39 (0.61)	
Secondary school	223	2.43 (0.92)		3.19 (0.35)		3.35 (0.29)		3.70 (0.52)	
Trade certificate	130	2.38 (0.92)		3.22 (0.37)		3.33 (0.29)		3.76 (0.56)	
Bachelor’s degree	172	2.51 (0.84)		3.40 (0.45)		3.40 (0.32)		3.75 (0.49)	
Post-graduate degree	126	2.44 (0.87)	0.467	3.43 (0.48)	**<0.001**	3.44 (0.28)	**0.003**	3.83 (0.63)	**0.043**
I prefer not to say	15	2.33 (0.72)		3.24 (0.42)		3.33 (0.28)		3.65 (0.48)	

Values for two non-binary respondents not shown due to small sample. *p* values < 0.05 bolded.

^a^*p* values from two-sample *t-*test (respondent status, gender, and vision loss status) or ANOVA (likelihood of taking up gene therapy, and education).

**Fig. 4:**
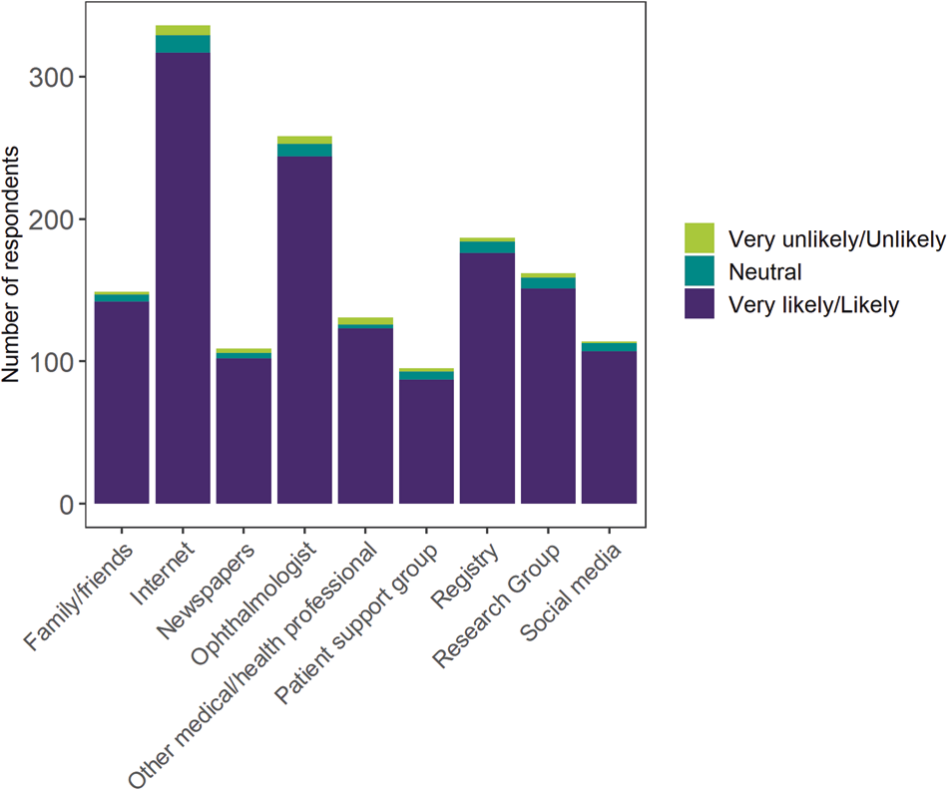
Information sources of people with IRD and their likelihood of accepting gene therapy (*n* = 681). Self-reported sources of information about inherited retinal disease compared with likelihood of accepting gene therapy of respondents to national survey of Australians with inherited retinal disease (total *n* = 681, adults with IRD *n* = 639, parents/caregivers *n* = 42) demonstrating that participants very likely to accept gene therapy if offered had sourced information from multiple sources, most commonly the internet (49%).

Respondents who indicated that they were likely/very likely to take up gene therapy scored higher on information sources (Subscale A; mean score 2.5 [SD: 0.90]), than people who were neutral (mean score 2.3 [0.82]) or unlikely/very unlikely to take up gene therapy (mean score 2.3 [0.89]), meaning that they are more likely to have obtained information about gene therapy from a greater number of sources (Fig. 4).

Overall, participants scored 3.3 out of 5 for knowledge of gene therapy methods, and 3.4 out of 5 for awareness of gene therapy outcomes. Mean subscale scores for Knowledge of Methods (Subscale B) were higher from respondents who indicated that they are likely/very likely to take up gene therapy (mean score 3.3 [0.42]) compared to those who were neutral (mean score 3.2 [0.36]) or unlikely/very unlikely to take up gene therapy (mean score 3.3 [0.53]; *p* = 0.036). Respondents with post-graduate degrees scored highest compared to other educational levels on Methods (*p* < 0.001) and Outcomes (*p* = 0.003).

Responses to items in the perceived value subscale (Subscale D) indicated that parents/caregivers were more likely to see value in gene therapy for their dependants than adults with IRD (mean subscale score 4.0 [0.44] vs. 3.7 [0.55], respectively, *p* = 0.002). Respondents who indicated that they are likely/very likely to take up gene therapy also scored higher for perceived values of gene therapy than those who were neutral or unlikely/very unlikely to take up gene therapy (mean scores 3.81 [0.54] vs. 3.60 [0.47] vs. 3.5 [0.59], respectively; *p* < 0.001). Respondents with post-graduate degrees were more likely to see economic value in treatment (*p* = 0.043).

### NEI-VFQ-25, PACT-22 and EQ-5D-5L

Scores of the NEI-VFQ-25, PACT-22 and EQ-5D-5L are shown in Supplementary Table S2. The median composite NEI-VFQ-25 score was 48 (IQR 38–62), from a range from 0 to 100. Responses to the PACT-22 showed high positive beliefs (mean score 95 [IQR: 81–100]), safety (mean score 88 [IQR: 75–94]), information needs (mean score: 88 [75–100]) and need for patient involvement (mean score 75 [69–88]). There were low negative expectations (mean score 42 [33–54]). Parents/caregivers were more likely to have high information needs (*p* = 0.035) and low negative expectations (*p* = 0.041) than adult patients with IRDs. Responses to the EQ-5D-5L showed overall utility score 0.81 and visual analogue score 77.

Comparing AGT-Eye subscale scores and other instruments, weak or no correlation was evident between AGT-Eye subscale scores and each of the NEI-VFQ-25 and EQ-5D-5L scores (Supplementary Table S3). For PACT-22 scores, weak positive correlations were observed between AGT-Eye knowledge of methods (subsection B) and awareness outcomes (subscale C) with PACT-22 positive beliefs (*ρ* = 0.11 and *ρ* = 0.11, respectively), safety (*ρ* = 0.10 and *ρ* = 0.09, respectively), and negative expectations (*ρ* = 0.20 and *ρ* = 0.11, respectively). For subsection D, weak correlations were observed between mean scores for AGT-Eye perceived value of treatment (Subscales C) and PACT-22 positive beliefs (*ρ* = 0.21), safety (*ρ* = 0.25), information needs (*ρ* = 0.16), and patient involvement (*ρ* = 0.21). A weak negative correlation was observed between AGT-Eye Information sources (Subsection A) and PACT-22 information needs (*ρ* = −0.11).

Comparing composite scores across the NEI-VFQ-25 and EQ-5D-5L instruments, a moderate correlation was observed between each of the NEI-VFQ-25 composite score with the EQ-5D-5L utility score (*ρ* = 0.57, 95% CI: 0.52–0.62) and the EQ-5D-5L visual analogue scale (*ρ* = 0.30, 95% CI: 0.23–0.37; Supplementary Table S4).

## Discussion

This is the first national Australian survey of the perspectives on gene therapy of people with IRDs (*n* = 639) and their parents/caregivers (*n* = 42). Based on current estimates of the prevalence of these conditions, we estimate that this sample represents ~5.3% of the Australian IRD population.

Respondents were consistent with previous international cohorts in terms of proportion with macular dystrophy phenotype [19, 20], reported NEI-VFQ visual disability [21–24] and health-related quality of life [25]. As expected, respondents with generalized IRD phenotypes reported more significant visual symptoms compared to macular dystrophy phenotypes, and were more likely to have used Vitamin A [26] as a treatment strategy.

We found that sources of information, knowledge of methods and outcomes of gene therapy for IRDs could be improved among potential recipients. Respondents were aware of these knowledge gaps, with only 28.3% agreeing that they “have good knowledge about gene therapy for inherited retinal diseases” (AGT-Eye item 1). “Good knowledge” was not defined in the survey questions or accompanying Plain Language Statement; we assumed it to mean respondents to have sufficient knowledge to make an informed decision about potentially receiving gene therapy for their IRD.

Despite awareness of knowledge gaps, on average, respondents placed value on the treatment and reported a willingness to undergo the treatment if it were available (91.6% likely or very likely). This willingness to access the relatively new treatment of gene therapy is comparable to prior findings in people with other systemic genetic conditions [7]. Knowledge gaps when new treatments are developed are to be expected, particularly by ophthalmologists when consenting people for novel genetic treatments.

Our novel tool, the AGT-Eye, provided useful information on the following themes:

1. Information sources (Subscale A): the internet was the main source of information about gene therapy for IRDs (49.3%), with low usage of other sources of information including social media (16.7%). Only 37.8% had received information from their ophthalmologist/s. This indicates a gap in education for potential recipients of these treatments [7–9]. Health care providers have also been shown to have a varying range of scientific knowledge of gene therapy [27–29], availability of services [29], and can be influenced by personal value-judgements about risk and benefit [30]. Genetic knowledge among ophthalmologists has not been assessed, but it is possible that low genetic knowledge of ophthalmologists contributed to their low usage as an information source. This subscale has highlighted the importance of targeted clinician education, as the rollout of ocular gene therapies continues. In addition, there is a role for high-quality online education sources, as around half of the respondents used this resource.
2. Knowledge of methods (Subscale B) and Awareness of outcomes (Subscale C): like Chapman et al. [31], we found participants generally had poor knowledge of the specific methods of retinal gene therapy, and that individuals with a higher level of education had a better understanding of the techniques, which is unsurprising given the technicality. Again, this subscale highlights the importance of education of both patients and caregivers (who may be asked to provide informed consent for clinical trials and approved genetic treatments).
3. Value of treatment (Subscale D): respondents generally saw value in treatment, with 79% agreeing that the government should subsidize treatment, 76.5% indicating a willingness to travel, and 62.3% indicating interest in a payment plan for treatment. Of note, these responses will be influenced by Australia’s hybrid health funding, with contributions to cost of care made by both government (about 70% via Federal universal insurance scheme and state-government administered hospitals) and recipients (about 30% via non-government sources including private health insurance) [32]. It is likely that responses to economic questions will vary in different jurisdictions.

The perceived high value of treatment suggests there may be a risk of therapeutic over-optimism or misconception [33]. This is suggested by respondents’ high participation in medical research in the past (28.3%), willingness to taking up gene therapy if available (91.6%) and PACT-22 responses which showed high positive beliefs, safety, and low negative expectations. Reasons for therapeutic optimism are generally thought to include low knowledge but may also include high level of trust in medical practitioners to provide suitable advice on safety and efficacy [34]. Others consider true optimism and belief in God to contribute [35]. Recommendations to reduce therapeutic misconception include further education of potential recipients of experimental treatments, providers, and the public [34].

Knowledge gaps are likely to continue increasing as new technologies develop. Closing these gaps will require continually evolving educational initiatives for both potential recipients of gene therapy and eyecare providers. Development of a core outcome set for ocular gene therapy by consensus between people with IRD and all other stakeholders will assist in identification of outcomes of importance to potential gene therapy recipients [36]. Together with results of further surveys of potential recipients, a core outcome set will more accurately delineate knowledge gaps, enabling development of targeted patient education materials. Our survey respondents favored the internet as a source of information; however, internet health information has been shown to be misleading [37], and further, diverse educational materials are recommended for optimum learning [38]. Developed educational initiatives will need to be patient-centered, culturally appropriate [39] and suitable for people with low vision [40]. Miesbach et al. describe a list of typical questions people with hemophilia may have before deciding to enter a gene therapy trial [41]. A modified list for ocular gene therapy could be a useful basis of educational initiatives for providers of gene therapy. Improved knowledge of gene therapy by both potential recipients and their ophthalmologists is fundamental to shared decision-making when gene therapy can be offered as a treatment option for IRDs [42, 43]. Improving clinician and patient education also has a role in treatment optimization for individuals, including decisions to not receive genetic therapies.

A key strength of this study was the large sample size, which came from a broad reaching recruitment campaign, largely through the assistance of Australian patient support groups. This enabled us to survey people from all over the country, including those who may have dropped out of regular eye-care, with a spectrum of IRDs and age, socioeconomic status etc. We ensured that the survey had multiple response options, all suitable for individuals with low vision (i.e., online, phone, paper-based). We also obtained responses from parents and caregivers, who are likely to be asked to provide informed consent for gene therapy of minors in the future. This ensures we are gathering perspectives from a variety of stakeholders.

Weaknesses of the study include recruitment of self-selected participants, which may skew results toward positive views of research and risk of therapeutic optimism. It is important to note that 28.3% of all respondents reported participation in medical research in the past, and 60.5% have supplied DNA to an Australian IRD database; comparison data with people with other genetic disease and the general IRD population is not available. Self-reported diagnosis of IRD may have been incorrect, either through misdiagnosis by the clinician or misunderstanding by the participant. The NEI-VFQ-25 is not optimized for persons with low vision from an IRD; patient related outcome tools for persons with IRD are in development, and will be of use in future studies of this nature [44]. The choice of AGT-Eye items and wording, with some negative responses required, may have contributed to the most common answer being “Neither agree nor disagree.” In contrast to other questions in subscale B (Knowledge of methods), item 1 assesses respondents’ self-reported knowledge of gene therapy. A response of having “good knowledge,” informs participants’ opinions rather than actual knowledge of gene therapy. Further discussion on the possible improvements of the AGT-Eye have previously been published [14]. Finally, it is not clear how the results may be extrapolated to other populations; Australia’s model of health care funding may have contributed to responses regarding value and funding of treatment.

Our study indicates knowledge gaps requiring further research in retinal gene therapy knowledge for potential gene therapy recipients, clinicians and the public; focusing on understanding gene therapy outcomes, risks and clinical research vs. approved treatments. The results of this study highlight the importance of both clinician and patient education, to counter knowledge gaps and lack of confidence in understanding of the treatment options. This is increasingly important due to the roll out of approved and experimental ocular gene therapy treatments. Finally, the survey revealed optimism and hope about emerging retinal gene therapies, with over 90% of respondents saying that they would be interested in receiving such a treatment. As the number of treatments increase, it is vital that education follows in line.

## Supplementary information

Supplementary material figure legend and tables

Supplementary Figure S1

Supplementary Table S3

## Data Availability

The dataset generated during and/or analyzed during the current study are available from the corresponding author on reasonable request.
