# Supplementary material for: Survey of perspectives of people with inherited retinal diseases on ocular gene therapy in Australia
Source: Gene Ther. 2022 Oct 2;30(3-4):336–46. doi: 10.1038/s41434-022-00364-z (PMC10113139; doi:10.1038/s41434-022-00364-z)
Supplement: Supplementary file 1 — Supplementary material figure legend and tables [file 41434_2022_364_MOESM1_ESM.docx]

Supplementary Figure 1: Response frequencies for Attitudes to Gene Therapy for the Eye items (n=681)

Supplementary Table S1: Visual status, previous treatments and presenting symptoms, by type of vision loss (n=681)

|  | **Type of vision loss** | | **Total** | **p-value*** |
| --- | --- | --- | --- | --- |
|  | **Generalized** | **Macular** |  |  |
|  | (n=525) | (n=156) | (N=681) |  |
| Age when symptoms first appeared (years) |  |  |  | **0.005** |
| Range | 0-80 | 0-81 | 0-81 |  |
| Median (IQR) | 20 (8-35) | 26 (11-44) | 20 (10-35) |  |
| Most recent decline in vision within, n (%) |  |  |  | 0.636 |
| No decline, stable vision | 53 (10.1%) | 22 (14.1%) | 75 (11.0%) |  |
| Less than 6 months | 65 (12.4%) | 17 (10.9%) | 82 (12.0%) |  |
| 1 Year | 107 (20.4%) | 31 (19.9%) | 138 (20.3%) |  |
| 5 Years | 180 (34.3%) | 48 (30.8%) | 228 (33.5%) |  |
| 10 Years | 120 (22.9%) | 38 (24.4%) | 158 (23.2%) |  |
| Past treatment for an IRD, n (%) |  |  |  |  |
| Acupuncture | 22 ( 4.2%) | 3 ( 1.9%) | 25 ( 3.7%) | 0.231 |
| Electrical stimulation | 10 ( 1.9%) | 1 ( 0.6%) | 11 ( 1.6%) | 0.471 |
| Human stem cells | 2 ( 0.4%) | 0 ( 0.0%) | 2 ( 0.3%) | >0.999 |
| Vitamin A | 92 (17.5%) | 11 ( 7.1%) | 103 (15.1%) | **0.001** |
| Herbal remedies | 51 ( 9.7%) | 27 (17.3%) | 78 (11.5%) | **0.009** |
| None of the above | 390 (74.3%) | 124 (79.5%) | 514 (75.5%) | 0.185 |
| Presenting symptoms for IRD, n (%) |  |  |  |  |
| Difficulty seeing at night or dusk | 401 (76.4%) | 61 (39.1%) | 462 (67.8%) | **<0.001** |
| Bumping into low lying objects | 253 (48.2%) | 23 (14.7%) | 276 (40.5%) | **<0.001** |
| Difficulty driving | 165 (31.4%) | 46 (29.5%) | 211 (31.0%) | 0.645 |
| Difficulty adjusting from light to dark and vice versa | 300 (57.1%) | 62 (39.7%) | 362 (53.2%) | **<0.001** |
| Missing parts in vision | 167 (31.8%) | 71 (45.5%) | 238 (34.9%) | **0.002** |
| Noticed peripheral or side vision reducing | 219 (41.7%) | 22 (14.1%) | 241 (35.4%) | **<0.001** |
| Other | 57 (10.9%) | 42 (26.9%) | 99 (14.5%) | **<0.001** |
| No noticeable symptoms | 7 ( 1.3%) | 8 ( 5.1%) | 15 ( 2.2%) | **0.005** |
| Can't recall | 16 ( 3.0%) | 7 ( 4.5%) | 23 ( 3.4%) | 0.382 |
| * p-value from Wilcoxon ranksum test (age), Fisher's exact test (acupuncture, electrical stimulation, stem cells, religious/personal tests), and Pearson's chi-squared tests for remaining variables.  + Multiple response options could be selected; analyzed as yes vs no for each option. | | | | |

Supplementary Table S2: Distribution of NEI-VFQ-25, PACT-22, and EQ-5D-5L instrument scores

|  | **Median (IQR)** | | | **p-value*** |
| --- | --- | --- | --- | --- |
|  | **Adult**  **patient** | **Parent/**  **caregiver** | **Total** |  |
|  |  |  |  |  |
|  | (n=639) | (n=42) | (N=681) |  |
| NEI-VFQ-25 total and subscales |  |  |  |  |
| Composite score | 48 (38-62) | 53 (38-68) | 48 (38-62) | 0.264 |
| General health | 75 (50-75) | 75 (50-100) | 75 (50-75) | <0.001 |
| General vision | 40 (20-60) | 40 (20-60) | 40 (20-60) | 0.908 |
| Ocular pain | 88 (63-100) | 75 (63-100) | 88 (63-100) | 0.150 |
| Near activities | 42 (17-67) | 45.8 (25-75) | 42 (17-67) | 0.326 |
| Distance activities | 42 (25-58) | 40 (25-67) | 42 (25-58) | 0.487 |
| Vision-specific social functioning | 50 (25-75) | 63 (38-88) | 50 (25-75) | 0.125 |
| Vision-specific mental health | 56 (38-75) | 50 (25-63) | 56 (38-69) | 0.012 |
| Vision-specific role difficulties | 38 (13-63) | 50 (25-75) | 38 (13-63) | 0.026 |
| Vision-specific dependency | 50 (17-75) | 46 (17-75) | 50 (17-75) | 0.418 |
| Driving | 0 (0-58) | 58 (58-92) | 0 (0-58) | 0.018 |
| Colour vision | 75 (50-100) | 50 (38-100) | 75 (50-100) | 0.181 |
| Peripheral vision | 25 (25-75) | 50 (25-75) | 25 (25-75) | 0.424 |
| PACT-22 dimensions |  |  |  |  |
| Positive beliefs | 93.75 (75-100) | 94 (81-100) | 94 (81-100) | 0.506 |
| Safety | 88 (75-94) | 88 (75-100) | 88 (75-94) | 0.217 |
| Information needs | 88 (75-100) | 94 (81-100) | 88 (75-100) | 0.035 |
| Negative expectations | 42 (33-54) | 42 (25-46) | 42 (33-54) | 0.041 |
| Patient involvement | 75 (69-94) | 75 (69-88) | 75 (69-88) | 0.944 |
| EQ-5D-5L |  |  |  |  |
| Utility score | 0.81 (0.68-0.90) | 0.80 (0.68-0.86) | 0.81 (0.68-0.90) | 0.382 |
| Visual analogue score | 77 (66-85) | 87 (76-93) | 77 (66-86) | <0.001 |
| NEI-VFQ-25 = National Eye Institute Visual Function Questionnaire  PACT-22 = Patient attitudes to clinical trials. * p-values from Wilcoxon rank-sum test | | | | |

Supplementary Table S3 provided separately in landscape format.

Supplementary Table S4. Spearman’s correlation between selected non-AGT-Eye instruments among adult patients

(n=639)

|  | **NEI-VFQ-25 total** | | **EQ-5D-5L utility score** | |
| --- | --- | --- | --- | --- |
|  | **ρ** | **(95% CI)** | **ρ** | **(95% CI)** |
| NEI-VFQ-25 total | 1.00 |  |  |  |
| EQ-5D-5L utility score | 0.57 | (0.52,0.62) | 1.00 |  |
| EQ-5D-5L VAS | 0.30 | (0.23,0.37) | 0.55 | (0.49,0.60) |
| VAS = visual analogue scale | | | | |
