# Supplementary figures and images for: Survey of perspectives of people with inherited retinal diseases on ocular gene therapy in Australia
Source: Gene Ther. 2022 Oct 2;30(3-4):336–46. doi: 10.1038/s41434-022-00364-z (PMC10113139; doi:10.1038/s41434-022-00364-z)

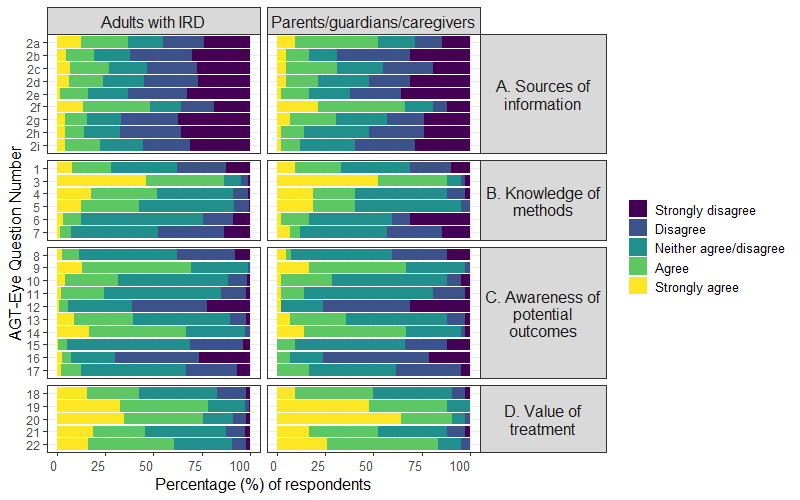

Supplement: Supplementary file 2 — Supplementary Figure S1 [file 41434_2022_364_MOESM2_ESM.tif]
