# Supplementary material for: Survey of perspectives of people with inherited retinal diseases on ocular gene therapy in Australia
Source: Gene Ther. 2022 Oct 2;30(3-4):336–46. doi: 10.1038/s41434-022-00364-z (PMC10113139; doi:10.1038/s41434-022-00364-z)
Supplement: Supplementary file 3 — Supplementary Table S3 [file 41434_2022_364_MOESM3_ESM.docx]

Supplementary Table S3: Spearman’s correlation between AGT-Eye subscale scores and other instruments among adult patients

(n=639)

|  | **AGT-Eye subscale** | | | | | | | |
| --- | --- | --- | --- | --- | --- | --- | --- | --- |
|  | **Information sources** | | **Methods** | | **Outcomes** | | **Value** | |
|  | **ρ** | **(95% CI)** | **ρ** | **(95% CI)** | **ρ** | **(95% CI)** | **ρ** | **(95% CI)** |
| NEI-VFQ-25 |  |  |  |  |  |  |  |  |
| Composite score | 0.02 | (-0.05,0.10) | 0.03 | (-0.05,0.11) | -0.01 | (-0.08,0.07) | 0.01 | (-0.06,0.09) |
| General health | 0.04 | (-0.03,0.12) | 0.12 | (0.04,0.19) | 0.07 | (-0.01,0.15) | 0.06 | (-0.02,0.14) |
| General vision | 0.03 | (-0.04,0.11) | 0.00 | (-0.08,0.08) | -0.01 | (-0.08,0.07) | -0.03 | (-0.11,0.05) |
| Ocular pain | -0.05 | (-0.13,0.03) | 0.01 | (-0.07,0.09) | 0.01 | (-0.06,0.09) | -0.04 | (-0.12,0.04) |
| Near activities | 0.03 | (-0.04,0.11) | 0.01 | (-0.07,0.09) | 0.01 | (-0.06,0.09) | 0.00 | (-0.07,0.08) |
| Distance activities | 0.05 | (-0.03,0.12) | 0.05 | (-0.03,0.12) | 0.02 | (-0.06,0.09) | 0.01 | (-0.07,0.08) |
| Vision-specific social functioning | 0.04 | (-0.04,0.12) | 0.04 | (-0.03,0.12) | -0.04 | (-0.12,0.04) | -0.04 | (-0.12,0.04) |
| Vision-specific mental health | -0.01 | (-0.08,0.07) | 0.00 | (-0.08,0.07) | 0.04 | (-0.04,0.12) | 0.10 | (0.03,0.18) |
| Vision-specific role difficulties | 0.02 | (-0.06,0.10) | 0.02 | (-0.06,0.10) | 0.00 | (-0.07,0.08) | -0.06 | (-0.13,0.02) |
| Vision-specific dependency | -0.03 | (-0.10,0.05) | -0.03 | (-0.10,0.05) | -0.02 | (-0.10,0.06) | 0.08 | (0.00,0.16) |
| Driving | 0.05 | (-0.04,0.14) | 0.01 | (-0.08,0.10) | -0.07 | (-0.15,0.02) | 0.01 | (-0.08,0.10) |
| Colour vision | -0.01 | (-0.08,0.07) | 0.04 | (-0.04,0.12) | 0.03 | (-0.05,0.10) | 0.04 | (-0.04,0.12) |
| Peripheral vision | -0.02 | (-0.10,0.05) | 0.04 | (-0.04,0.11) | -0.04 | (-0.12,0.04) | -0.02 | (-0.09,0.06) |
| PACT-22 |  |  |  |  |  |  |  |  |
| Positive beliefs | -0.03 | (-0.11,0.05) | 0.11 | (0.03,0.18) | 0.11 | (0.03,0.18) | 0.21 | (0.14,0.28) |
| Safety | 0.04 | (-0.04,0.12) | 0.10 | (0.02,0.17) | 0.09 | (0.01,0.16) | 0.25 | (0.18,0.32) |
| Information needs | -0.11 | (-0.19,-0.04) | -0.01 | (-0.09,0.06) | 0.04 | (-0.03,0.12) | 0.16 | (0.09,0.24) |
| Negative expectations | 0.00 | (-0.08,0.08) | 0.20 | (0.12,0.27) | 0.11 | (0.03,0.19) | -0.08 | (-0.15,0.00) |
| Patient involvement | 0.02 | (-0.06,0.10) | 0.03 | (-0.04,0.11) | 0.02 | (-0.06,0.10) | 0.20 | (0.13,0.27) |
| EQ-5D-5L |  |  |  |  |  |  |  |  |
| Utility score | 0.04 | (-0.03,0.12) | 0.03 | (-0.05,0.11) | -0.06 | (-0.14,0.02) | -0.05 | (-0.12,0.03) |
| Visual analogue score | -0.01 | (-0.09,0.06) | -0.01 | (-0.08,0.07) | -0.02 | (-0.09,0.06) | 0.00 | (-0.08,0.07) |
| Positive Spearman’s correlations shaded green, negative Spearman’s correlations shaded blue (when p<0.05). | | | | | | | | |
